# Large Language Models–Supported Thrombectomy Decision-Making in Acute Ischemic Stroke Based on Radiology Reports: Feasibility Qualitative Study

**DOI:** 10.2196/48328

**Published:** 2025-02-13

**Authors:** Jonathan Kottlors, Robert Hahnfeldt, Lukas Görtz, Andra-Iza Iuga, Philipp Fervers, Johannes Bremm, David Zopfs, Kai R Laukamp, Oezguer A Onur, Simon Lennartz, Michael Schönfeld, David Maintz, Christoph Kabbasch, Thorsten Persigehl, Marc Schlamann

**Affiliations:** 1 Institute for Diagnostic and Interventional Radiology Faculty of Medicine and University Hospital Cologne University of Cologne Cologne Germany; 2 Department of Neurology Faculty of Medicine and University Hospital Cologne University of Cologne Cologne Germany

**Keywords:** artificial intelligence, radiology, report, large language model, text-based augmented supporting system, mechanical thrombectomy, GPT, stroke, decision-making, thrombectomy, imaging, model, machine learning, ischemia

## Abstract

**Background:**

The latest advancement of artificial intelligence (AI) is generative pretrained transformer large language models (LLMs). They have been trained on massive amounts of text, enabling humanlike and semantical responses to text-based inputs and requests. Foreshadowing numerous possible applications in various fields, the potential of such tools for medical data integration and clinical decision-making is not yet clear.

**Objective:**

In this study, we investigate the potential of LLMs in report-based medical decision-making on the example of acute ischemic stroke (AIS), where clinical and image-based information may indicate an immediate need for mechanical thrombectomy (MT). The purpose was to elucidate the feasibility of integrating radiology report data and other clinical information in the context of therapy decision-making using LLMs.

**Methods:**

A hundred patients with AIS were retrospectively included, for which 50% (50/100) was indicated for MT, whereas the other 50% (50/100) was not. The LLM was provided with the computed tomography report, information on neurological symptoms and onset, and patients’ age. The performance of the AI decision-making model was compared with an expert consensus regarding the binary determination of MT indication, for which sensitivity, specificity, and accuracy were calculated.

**Results:**

The AI model had an overall accuracy of 88%, with a specificity of 96% and a sensitivity of 80%. The area under the curve for the report-based MT decision was 0.92.

**Conclusions:**

The LLM achieved promising accuracy in determining the eligibility of patients with AIS for MT based on radiology reports and clinical information. Our results underscore the potential of LLMs for radiological and medical data integration. This investigation should serve as a stimulus for further clinical applications of LLMs, in which this AI should be used as an augmented supporting system for human decision-making.

## Introduction

Acute ischemic stroke (AIS) is a major cause of death and disability, requiring effective and efficient therapeutic strategies. To prevent the progression of ischemic damage and improve patient outcomes, timely and accurate diagnosis and treatment are crucial [1-3]. The standard of care and diagnostic workflow for acute AIS involves a combination of clinical information, clinical assessment, imaging, and decision-making tools, such as the National Institutes of Health Stroke Scale (NIHSS) [2,4-6]. Current treatment approaches include the administration of intravenous thrombolytic therapy, as well as mechanical thrombectomy (MT), either as a combined or sole treatment option [5,7,8]. While MT is an invasive procedure aiming for the removal of an occluding thrombus, only patients fulfilling certain requirements are eligible for this specific treatment [9,10]. The indication for MT is established with a combination of clinical assessment, image-based results, and guidelines such as the European Society of Neuroradiology or European Stroke Organization guidelines, taking into account factors such as patient’s age, onset time of symptoms, and location and extent of the occlusion [8,10,11].

However, the subjective nature of the clinical assessment and decision-making tools, combined with the growing workload of health care professionals, highlights the need for a supporting, objective, and efficient approach to decision-making [12]. The task of deciding a patient’s suitability for MT can be particularly challenging for inexperienced physicians, as it requires a thorough understanding of the latest medical evidence and guidelines, as well as a high degree of clinical judgment [13-15].

Studies have demonstrated the feasibility and potential of using artificial intelligence (AI) in medical decision-making, particularly in the radiology field [16,17]. For example, recent studies show the application of AI in cancer imaging analysis or in detecting acute intracranial hemorrhage on computed tomography (CT) or magnetic resonance imaging scans [18,19]. Similar studies have also shown promising results in the use of AI in the diagnosis of ischemic stroke (IS), with potential improvement of diagnostic accuracy and reduction of variability in the decision-making process. However, these studies all focus on the AI-based processing of visual information [18,20].

As nearly all information can be expressed in human language, recorded in text form in the radiology report, recent advancements in the development of large language models (LLMs) have opened new opportunities for medical information processing [21-24]. One of these autoregressive LLMs is GPT-3 used within the pretrained chatbot application version, named ChatGPT, developed by OpenAI [25]. GPT-3 is a third-generation deep learning model that has been trained on a massive amount of text data on 175 billion parameters, allowing it to generate humanlike logical and semantical responses to text-based questions and input information [26,27]. In an experimental setting, a GPT-3–based LMM model shows a significant improvement in medical question-answering tasks and showed a passing score in a medical licensing examination, due to its ability to provide logical and informational context across most answers [28]. To our knowledge, no large language processing model has been evaluated for immediate clinical decision-making based on radiology report texts. This could carry several benefits, including increasing the objectivity, speed, and standardization of decisions, as well as assisting physicians and medical staff by highlighting or reminding them of potential courses of action based on the latest medical evidence and guidelines.

In the context of AIS diagnosis and treatment, the application of such an LLM to a radiology report with a resulting decision-making component related to treatment using MT represents an exemplary use case. In this way, possible AI-assisted decisions related to MT could be made faster and more standardized based on up-to-date medical evidence and guidelines, which can be checked and correlated to the radiology report. Additionally, the application of an LLM as a monitoring, AI-augmented background system, supporting inexperienced or younger radiologists by highlighting the potential MT indication based on the written report, is conceivable. Thus, this study aims to evaluate the accuracy and efficiency of LLMs in predicting the suitability of patients for MT and to compare its decisions with those made in consensus by experienced health care professionals.

## Methods

### Ethical Considerations

This retrospective study received human subject ethics review approvals (23-1061-retro). Informed consent was waived due to the retrospective design of this investigation including the ability of participants to opt out. Privacy and confidentiality were ensured—beyond the patient’s age and sex, no identifiable features about the patient were transmitted to the LLM model; especially, no patient-identifying information was provided to the AI.

### Patient Population

The inclusion criteria for this study were (1) clinical symptoms consistent with AIS; (2) evaluable CT scan according to in-house stroke protocol and board-certified radiology report; (3) availability of the NIHSS score; (4) presentation to the hospital within a potential time of treatment options including MT; (5) for group affiliation, the decision to perform MT was evaluated based on an interdisciplinary expert consensus immediately before MT in each case and classified into (a) indication of MT and (b) no indication of MT; and (6) aged 18 years or older.

The exclusion criteria for this study were (1) patients in whom the juristic decision-making authority or written intention potentially influences the decision to undergo MT and (2) significant comorbidities that would limit the ability to undergo MT or the ability to provide informed consent.

### Workflow

**Figure 1:**
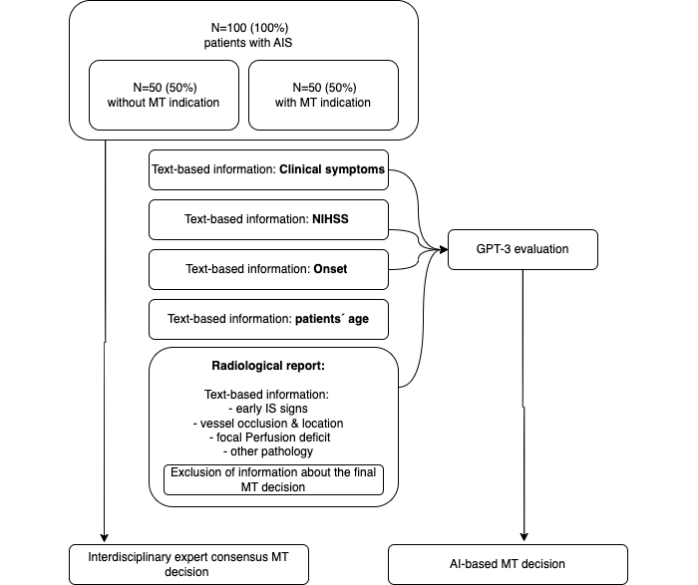
A hundred patients with AIS were analyzed. The patients were divided into two groups: 50% (50/100) had an indication for MT and the other 50% (50/100) had no MT indication. The image information, in the form of the radiology report, was transmitted to the AI, together with additional information on NIHSS, neurological symptoms, symptoms onset, and patients’ age. However, the final decision regarding MT was not transmitted to the AI. The text-based AI was asked whether there was an indication for MT. The AI decision was compared with the decision of the health care professional’s. AI: artificial intelligence; AIS: acute ischemic stroke; IS: ischemic stroke; MT: mechanical thrombectomy; NIHSS: National Institutes of Health Stroke Scale.

The Institute for Diagnostic and Interventional Radiology University Hospital Cologne performs about 250 intracranial MT per year. We retrospectively screened the database according to the abovementioned inclusion criteria in the period between January 2020 to December 2022 and randomly selected a total of 100 patients with AIS, whereby 50% (50/100) patients have an MT indication and 50% (50/100) patients have no MT indication. All patients were eligible for a priori consideration of MT, the NIHSS was assessed before CT imaging, and neurological symptoms were recorded. The onset of neurological symptoms was also recorded based on case history, including unknown time frames. The image information, in the form of the radiology report, was transmitted to the AI, together with additional information on neurological symptoms, symptoms onset, and patients’ age, but the final decision regarding MT was not transmitted to the AI (Figure 1). Based on 20% (20/100) of the cases, we performed a test re-evaluation by the LLM.

### Neurological Assessment

A comprehensive neurological assessment was performed on all patients by a trained neurologist before the radiological examination. This assessment included the documentation of the NIHSS score including neurological symptoms and their onset, which was then communicated as part of the patient’s medical history included in the request for radiologic imaging.

### Imaging Protocol

All examinations used 128-254 slice CT scanners. Patients were scanned when supine from head to toe. The protocol included an unenhanced neurocranium CT and arterial phase imaging of cervical and intracranial vessels after injecting 60 mL of contrast media (Accupaque 350 mg/mL) at 4 mL/s, followed by 20 mL saline. Bolus tracking activated a 3-second scan delay after reaching 150 Hounsfield units in the descending thoracic aorta. The settings were pitch 1, 120 kVp, and 512 matrix with the DoseRight 3D-DOM (Philips Healthcare). Images were reconstructed axially with a 0.67-mm slice thickness. In some cases, CT perfusion was carried out with an additional 40 mL contrast agent and 20 mL saline using an 80-kVp, 512-matrix DoseRight 3D-DOM and processed with Philips IntelliSpace software (Philips Healthcare).

### Image Analysis

In the study, 12 assistant radiologists (2-4 years’ experience in neuroradiological imaging) were primarily responsible for conducting the primary image analysis. The radiologic findings were then secondarily reviewed and evaluated in an interdisciplinary consensus with neuroradiologists and neurologists, who had an average experience of over 5 years in neuroradiological imaging. The final decision regarding MT was reached based on this interdisciplinary consensus.

### Radiology Report

The text information in the form of a narrative, text-based radiology report in the German language included various imaging findings related to the presence of an AIS. This information was recorded as a prose text. The radiological findings were transmitted to the LLM together with the patient’s age and respective NIHSS score, neurologic symptomatology, and the corresponding time windows (time between onset of symptomatology and time of CT scan). Any information on the final MT decision was not included. No other personal data were transmitted to the LLM during the process. The average word count per report was 186 (SD 14; range 113-278).

### LLM-Based Decision-Making

GPT-3 is a commercial-sector, pretrained, state-of-the-art, language-processing AI model developed by OpenAI, which is known for its ability to assess text information and has been seen as a significant advancement in natural language processing technology [27]. It has been trained on a massive amount of text data obtained from the internet and has been fine-tuned for various language tasks such as text completion, translation, and question answering. The used LLM model is specifically designed for conversational AI applications, such as virtual assistants [27].

In this study, GPT-3 in the form of ChatGPT was used for information processing and decision-making tasks. The text-based information received was used to generate a decision response. As described above, the information from the radiology report as well as clinical information and patients’ ages were provided to the LLM, and the following request was made to it to provide a decision [29]:

Please decide whether mechanical thrombectomy is indicated or not, based on the following data. Please answer yes or no.

### Statistical Analysis

Statistical data analysis was performed using R (version 3.6.2; R Foundation for Statistical Computing) on RStudio (version 1.2.5033.35; R Foundation). Figures were plotted using the *ggplot2* package. Continuous variables were reported as mean and SD. Demographic characteristics were compared using the chi-square test for categorical variables and 2-tailed *t* test for continuous variables. The diagnosis’ overall accuracy, specificity, and sensitivity were assessed using contingency tables.

## Results

### Baseline Characteristics

The demographic data were collected and analyzed for a total of 100 patients with AIS, who were divided into two groups: group 1 (n=50, 50%) received MT treatment, while group 2 (n=50, 50%) did not receive MT treatment. The average age of the participants was 68.34 (SD 6.95) years, with no significant difference between the two groups (*P*=.32). The distribution of sex was 54% (27/50) male in group 1 and 46% (23/50) in group 2 (*P*=.82). There were no significant differences between the two groups according to the demographic data (Table 1).

**Table 1:** Baseline characteristics of this study’s population, presented by treatment group. The table displays the mean age (SD), sex distribution (male and female), and NIHSS^a^ score and time from clinical onset to imaging (SD) for two treatment groups. Group 1 received MT^b^, while group 2 did not receive MT.

Group	Age (years), mean (SD)	Sex, n (%)			NIHSS score, mean (SD)		Onset to imaging (min)
		Male	Female				
Group 1 (MT; n=50)	67.44 (8.34)	27 (54)	23 (46)	14.28 (2.21)		93 (50^c^)	
Group 2 (no MT; n=50)	69.23 (5.56)	23 (46)	27 (54)	9.22 (2.71)		129 (113^d^)	

^a^NIHSS: National Institutes of Health Stroke Scale.

^b^MT: mechanical thrombectomy.

^c^Unknown onset of symptoms in 8 cases.

^d^Unknown onset of symptoms in 14 cases.

Regarding the NIHSS scores, the average score was found to be higher in group 1 (14.28, SD 2.21) than in group 2 (9.22, SD 2.71), suggesting that the patients in group 1 had more severe IS symptoms than those in group 2. The difference between the two groups was statistically significant (*P*=.02; Table 1). In the MT group, the time from the clinical onset of symptoms to CT imaging was on average 93 (SD 50) minutes (unknown onset of symptoms in n=8 cases), while in the no-MT indication group, the time was 129 (SD 113) minutes (unknown onset of symptoms in n=14 cases). There was no significant difference between the two groups (*P*=.051; Table 1).

In both groups, the most common symptoms were paresis and aphasia. In the group with MT indication, 72% (36/50) of patients presented with paresis and 46% (23/50) of patients presented with aphasia. In the group without MT indication, 76% (38/50) of patients presented with paresis and 26% (13/50) of patients presented with aphasia. Anopsia was less frequent, with only 2% (1/50) of patients in the MT group and 6% (3/50) of patients in the group without MT indication. Loss of consciousness was also less common, with 18% (9/50) of patients in the MT group and 4% (2/50) of patients in the group without MT indication (Table 2).

**Table 2:** Frequency and percentage of the most common clinical symptoms observed before imaging in two groups.

Group	Paresis, n (%)	Aphasia, n (%)	Anopsia, n (%)	Loss of consciousness, n (%)
Group 1 (MT^a^; n=50)	36 (72)	23 (46)	1 (2)	9 (18)
Group 2 (no MT; n=50)	38 (76)	13 (26)	3 (6)	2 (4)

^a^MT: mechanical thrombectomy.

### Performance of AI Decision-Making

The decision-making process of the AI-based model was compared to the interdisciplinary consensus regarding MT. The specificity, sensitivity, and accuracy of the AI-based model were calculated based on the agreement with the expert consensus. The results showed that the AI had a specificity of 0.96, a sensitivity of 0.8, and an accuracy of 0.88 in comparison to the expert consensus (Table 2). The area under the curve for report-based MT decision was calculated to be 0.92, indicating good discrimination between the two treatment options (Table 3). The re-evaluation of 20% (20/50) of the cases showed a 100% (20/20) concordance between the results of the first and second LLM evaluations.

**Table 3:** Number of true positive, false negative, true negative, and false positive cases for two groups: group 1 (MT^a^) and group 2 (no MT).

Group	True positive, n	False negative, n	True negative, n	False positive, n
Group 1 (MT)	40	10	—^b^	—
Group 2 (no MT)	—	—	48	2

^a^MT: mechanical thrombectomy.

^b^Not applicable.

## Discussion

This investigation assesses a new approach applying a text-processing AI model to the decision-making process for MT indication in IS, primarily based on the information from the radiology report. Descriptive information on the radiological findings, along with information on patients’ neurological symptoms, symptom onset, and demographic data, were provided to the LLM to evaluate the possibility of MT.

This study compared the AI-based decision-making process with the clinical interdisciplinary consensus for MT. Although the accuracy was good, the specificity of the AI model was lower than the sensitivity, with a specificity of 96% and a sensitivity of 80%. The overall accuracy of the AI-based model was 88%, indicating a good discrimination between the two treatment options, with an area under the curve of 0.92 for the AI report–based MT decision.

One of the strengths of this study is the robust gold truth: the well-defined interdisciplinary performed expert consensus for or against MT. Additionally, all cases were evaluated based on radiographic findings, neurological diagnostic information, as well as demographic data on the patient [10]. The AI model used in this study was not specifically trained for this task of making a clinical decision, being rather a language processing algorithm that considers a large amount of human declarative and conceptual knowledge up until 2021.

However, there are several limitations to this study. First, only text-based information was included in the decision-making process, and descriptive statements can be inaccurate and lack quantitative precision. For example, the adjective “large” can have varying interpretations between individuals, which potentially can lead to misinterpretation of the decision and result in AI decision consequences. Second, as the LLM model only has knowledge up until 2021, newer guidelines and developments in the field are not considered [27]. Third, the basis of a decision made by a model is not deterministically understandable and post hoc traceable, which represents a fundamental “black-box” problem of decisions based on structures of deep neural networks [12]. Moreover, within LLM, there is no possibility to show sources and retrieve information about the source of a decision. Further, and this is a fact that may be attributed to the commercial structure of OpenAI’s GPT-3, although the algorithm has been trained on vast amounts of text data, it remains unclear which data have been included or excluded [12,26,27,30]. In particular, the possibility that potentially older outdated information that would not have resulted in an MT indication and has been made available to the algorithm at an unspecified location for training purposes could lead to problems. Another limitation pertains to the fact that the algorithm can only consider general information for the MT decision, without considering individual factors about the individual health care facility or medical personnel. Factors such as (1) availability of material for MT, (2) personnel or time capacities, and (3) personal skills of the interventionist are not part of the MT decision [14]. Experimental settings and individual treatment attempts are not part of a potential LLM-based decision. Another limitation is that only text-based reports from one center in one language were included; further studies should include radiological findings from other centers with diverse linguistic styles and also other languages.

In particular, the described facts that current data and information, as well as the individual skills of the interventionalist, could be considered in the decision-making process may explain the lower sensitivity of the AI-based MT decision in relation to its specificity.

In summary, there are several reasons why AI should not make important medical decisions such as MT decisions on its own. Nonetheless, a decision-making algorithm based on the written radiology report can potentially be used to run in the background and alert to a potential MT indication. A potential application would be in the context of an augmenting system that “monitors” radiology reports of younger or inexperienced physicians in the background and alerts in specific cases so that an experienced colleague can be consulted for the final decision. This integration of augmented text-based AI could be especially useful in night shifts, as it has the potential to shorten the time from diagnosis to MT (door-to-needle time). In summary, this could lead to a sustainable improvement in patient care and apply to other areas beyond interventional neuroradiology.

Future studies should consider incorporating additionally other AI networks, such as deep learning–based image recognition networks, to make decisions based on more quantitative information, rather than just on declarative text information. This could provide more objective information, such as the amount of brain tissue that can be saved, which would help to further refine the decision-making process. In the context of further research, it would be preferable that the algorithm specifies the exact source or guideline on which the decision is based, highlighting the need for stable, finalized, and specifically tuned systems for particular tasks in clinical settings. For clinical use of text-based advanced assist for radiologists, quantitative and qualitative information about the AI training data used should be available. Additionally, future studies should also consider incorporating more recent information and developments in the field, as well as available MT devices and the experience of the treating interventionalist.

In conclusion, this study highlights within a first application the potential of LLMs for integrating text-based radiological information with other clinical data, which may be useful for augmented clinical decision-making. Further research should elucidate the limitations of such systems, particularly regarding the traceability of related training and reference data.

## Abbreviations

AI: artificial intelligence
AIS: acute ischemic stroke
CT: computed tomography
IS: ischemic stroke
LLM: large language model
MT: mechanical thrombectomy
NIHSS: National Institutes of Health Stroke Scale
